# Supplementary material for: Cardiovascular disease risk factors induce mesenchymal features and senescence in mouse cardiac endothelial cells
Source: eLife. 2021 Mar 4;10:e62678. doi: 10.7554/eLife.62678 (PMC8043751; doi:10.7554/eLife.62678)
Supplement: Figure 1—source data 2. [file elife-62678-fig1-data2.pdf]

Figure 1- Figure supplement 4. Echocardiography measurements of cardiac function and ventricular dimensions.

Experiment groups	IVS,d (mm)	IVS,s (mm)	LVID,d (mm)	LVID,s (mm)	LVPW,d (mm)	LVPW,s (mm)	LV Vol,d (µl)	LV Vol,s (µl)	LV mass (mg)	FS (%)
6 weeks of treadmill running										
**EXE (n=5); 3 mo old**	1.02±0.14	1.33±0.13	3.84±0.10	2.56±0.12†	0.74±0.05	1.25±0.05	63.8±4.01	23.9±2.73†	103.6±19.1	33.5±2.3
**SED (n=5); 3 mo old**	0.89±0.07	1.13±0.11	3.93±0.05	2.94±0.10	0.89±0.09	1.22±0.13	67.2±1.85	33.5±2.83	106.9±4.4	25.3±1.9
14 weeks of high fat diet feeding										
**HFD (n=5); 3 mo old**	1.11±0.04†	1.39±0.06	3.92±0.13	3.10±0.21	0.86±0.03†	1.09±0.08	67.2±5.32	39.3±6.5	121.7±5.2	21.1±3.3†
**CHOW (n=5); 3 mo old**	0.93±0.05	1.41±0.03	4.19±0.08	2.96±0.08	0.75±0.04	1.05±0.15	78.5±3.54	34.2±2.4	107.9±3.8	29.4±1.6
18 months of aging										
**AGED (n=6); 18 mo old**	1.05±0.03†	1.39±0.06†	4.16±0.11	3.31±0.13†	1.09±0.06†	1.34±0.06†	77.2±4.4	44.9±4.1 †	150.1±9.1†	20.7±1.6†
**YOUNG (n=6); 2 mo old**	0.85±0.04	1.19±0.06	3.94±0.10	2.84±0.13	0.77±0.06	1.13±0.03	68.1±4.4	31.0±3.3	93.8±3.9	27.9±1.7
Trans-aortic constriction: Week 0-6										
**Sham(n=5);Week 0**	0.78±0.03	1.12±0.03	3.50±0.06	2.31±0.05	0.79±0.01	1.19±0.03	51.0±2.0	18.2±1.2	73.5±1.6	34.1±0.8
**TAC (n=10);Week 0**	0.77±0.03	1.10±0.04	3.67±0.12	2.57±0.13	0.81±0.02	1.20±0.03	58.1±5.0	24.8±3.6	80.1±3.1	30.4±1.3
**Sham(n=5);Week 1**	0.73±0.03	1.07±0.06	3.88±0.09	2.74±0.12	0.75±0.03	1.09±0.05	65.4±3.7	28.4±3.1	80.1±2.2	29.4±1.9
**TAC (n=10);Week 1**	0.87±0.04†	1.18±0.06	3.92±0.12	3.03±0.18	0.98±0.06†	1.25±0.07	67.6±5.1	37.8±5.0	111.6±7.1†	23.2±2.3
**Sham(n=5);Week 2**	0.70±0.04	1.07±0.05	3.89±0.06	2.63±0.07	0.76±0.02	1.11±0.04	65.6±2.8	25.6±1.8	79.3±3.2	32.4±1.4
**TAC (n=10);Week 2**	0.87±0.04†	1.14±0.05	4.12±0.15	3.34±0.21†	1.02±0.03†	1.27±0.04†	76.9±7.3	48.0±7.4 †	124.4±6.2†	19.5±2.6†
**TAC (n=5); Week 3**	0.97±0.04†	1.17±0.04	4.38±0.19†	3.77±0.16	1.08±0.06†	1.29±0.07	87.8±9.8	61.4±6.6 †	153.6±12.2†	13.8±0.9†
**TAC (n=5); Week 4**	0.92±0.05†	1.20±0.06	4.60±0.23†	4.04±0.24	1.25±0.10†	1.37±0.09	99.0±13 †	72.8±11 †	178.3±9.9	12.4±0.8†
**TAC (n=5); Week 5**	0.89±0.10†	1.15±0.11	4.92±0.07†	4.40±0.09	1.00±0.05†	1.12±0.03†	114.0±4 †	87.8±4.3 †	165.6±10	10.6±1.4†
**TAC (n=5); Week 6**	0.81±0.06	0.96±0.08	5.15±0.22†	4.82±0.24	0.98±0.09†	1.01±0.09	127.8±13†	110.2±13†	164.9±7.9	6.4±0.8 †

mo: Months; **IVS,d:** Interventricular septum thickness at end-diastole; **IVS,s:** Interventricular septum thickness at end-systole; **LVID,d:** Left ventricular internal dimension at end-diastole; **LVID,s:** Left ventricular internal dimension at end-systole; **LVPW,d:** Left ventricular posterior wall thickness at end-diastole; **LVPW,s:** Left ventricular posterior wall thickness at end-systole; **LV Vol, d:** Left ventricular volume at end-diastole; **LV Vol,s:** Left ventricular volume at end-systole; **LV mass:** Left ventricular mass; **FS:** Fractional shortening.

Data are mean±SEM. Student *t* test was used, † $p<0.05$.
