# Supplementary material for: Cardiovascular disease risk factors induce mesenchymal features and senescence in mouse cardiac endothelial cells
Source: eLife. 2021 Mar 4;10:e62678. doi: 10.7554/eLife.62678 (PMC8043751; doi:10.7554/eLife.62678)
Supplement: Figure 3—source data 1. — (A) Reference list for endothelial and mesenchymal genes indicated in the Figure 3B (EXE vs. SED) heat map. (B) Reference list for endothelial and mesenchymal genes indicated in the Figure 3C (aged vs. young) heat map. (C) Reference list for endothelial and mesenchymal genes indicated in the Figure 3D (high-fat diet [HFD] vs. Chow) heat map. (D) Reference list for endothelial and mesenchymal genes indicated in the Figure 3E (transverse aortic constriction [TAC] [2] vs. Sham) heat map. (E) Reference list for endothelial and mesenchymal genes indicated in the Figure F (TAC [7] vs. Sham) heat map. [file elife-62678-fig3-data1.docx]

**Figure 3 – Source data 1: Reference list for endothelial and mesenchymal genes indicated in the Figure 3B (EXE vs. SED) heat map.**

| Gene | Description | Reference # |
| --- | --- | --- |
| *Malat1* | Metastasis Associated Lung Adenocarcinoma Transcript 1 | ^1^ |
| *Mgp* | Matrix Gla Protein | ^2^ |
| *Ankrd11* | Ankyrin Repeat Domain 11 | ^3^ |
| *Plcb4* | Phospholipase C Beta 4 | ^4^ |
| *Rock1* | Rho Associated Coiled-Coil Containing Protein Kinase 1 | ^5^ |
| *Adgrg6* | Adhesion G Protein-Coupled Receptor G6 | ^6^ |
| *Ppp4r3a* | Protein Phosphatase 4 Regulatory Subunit 3A | ^7^ |
| *Phip* | Pleckstrin Homology Domain Interacting Protein | ^8^ |
| *Clk1* | CDC Like Kinase 1 | ^9^ |
| *Ankrd12* | Ankyrin Repeat Domain 12 | ^10^ |
| *Ppl* | Periplakin | ^11^ |
| *Krit1* | KRIT1 Ankyrin Repeat Containing | ^12, 13^ |
| *Calcrl* | Calcitonin Receptor Like Receptor | ^14^ |
| *Apln* | Apelin | ^15^ |
| *Aplnr* | Apelin Receptor | ^16^ |
| *Vash1* | Vasohibin 1 | ^17, 18^ |
| *Fscn1* | Fascin Actin-Bundling Protein 1 | ^19^ |
| *Cd93* | CD93 Molecule | ^20, 21^ |
| *Vwa1* | Von Willebrand Factor A Domain Containing 1 | ^22, 23^ |
| *Adamts4* | ADAM Metallopeptidase With Thrombospondin Type 1 Motif 4 | ^24^ |
| *Sparc* | Secreted Protein Acidic And Cysteine Rich | ^25^ |
| *Col4a2* | Collagen Type IV Alpha 2 Chain | ^26, 27^ |
| *Cd34* | CD34 Molecule | ^28, 29^ |
| *Tuba1a* | Tubulin Alpha 1a | ^30^ |
| *Cx3cl1* | C-X3-C Motif Chemokine Ligand 1 | ^31^ |
| *Mest* | Mesoderm Specific Transcript | ^32^ |
| *Cnn2* | Calponin 2 | ^33^ |
| *Cd44* | CD44 Molecule (Indian Blood Group) | ^34^ |
| *Trp53* | Tumor Protein P53 | ^35^ |
| *Prex1* | Phosphatidylinositol-3,4,5-Trisphosphate Dependent Rac Exchange Factor 1 | ^36^ |
| *Hspg2* | Heparan Sulfate Proteoglycan 2 | ^37^ |
| *Tnfaip1* | TNF Alpha Induced Protein 1 | ^38^ |
| *Lamb1* | Laminin Subunit Beta 1 | ^39^ |
| *Ltbp4* | Latent Transforming Growth Factor Beta Binding Protein 4 | ^40^ |
| *Sept4* | Septin 4 | ^41^ |
| *Unc5b* | Unc-5 Netrin Receptor B | ^42^ |

**Figure 3 – Source data 1:** Reference list for endothelial and mesenchymal genes indicated in the Figure 3C (Aged vs. Young) heat map.

| Gene | Description | Reference # |
| --- | --- | --- |
| *Npr3* | Natriuretic Peptide Receptor 3 | ^43, 44^ |
| *Tagln2* | Transgelin 2 | ^45^ |
| *Vim* | Vimentin | ^46^ |
| *Msn* | Moesin | ^47-49^ |
| *Socs5* | Suppressor Of Cytokine Signaling 5 | ^50^ |
| *Vcam1* | Vascular adhesion molecule 1 | ^51^ |
| *Notch4* | Notch Receptor 4 | ^29, 52^ |
| *Stat6* | Signal Transducer And Activator Of Transcription 6 | ^53^ |
| *Adamtsl4* | ADAMTS Like 4 | ^54^ |
| *Jag2* | Jagged Canonical Notch Ligand 2 | ^29^ |
| *Cdh13* | Cadherin 13 | ^55, 56^ |
| *Hif1a* | Hypoxia Inducible Factor 1 Subunit Alpha | ^29^ |
| *Ism1* | Isthmin 1 | ^57, 58^ |
| *Tbx3* | T-Box Transcription Factor 3 | ^59^ |
| *Tgfa* | Transforming Growth Factor Alpha | ^60^ |
| *Furin* | Furin, Paired Basic Amino Acid Cleaving Enzyme | ^61^ |
| *Eng* | Endoglin | ^62, 63^ |
| *Smad6* | SMAD Family Member 6 | ^64, 65^ |
| *Smad7* | SMAD Family Member 7 | ^64, 65^ |
| *Sox17* | SRY-Box Transcription Factor 17 | ^66^ |
| *Tead1* | TEA Domain Transcription Factor 1 | ^29, 67^ |
| *Tead2* | TEA Domain Transcription Factor 2 | ^29,67^ |
| *Klf3* | Kruppel Like Factor 3 | ^68, 69^ |
| *Klf4* | Kruppel Like Factor 4 | ^70^ |
| *Sulf1* | Sulfatase 1 | ^71^ |
| *Wt1* | WT1 Transcription Factor | ^72, 73^ |
| *Ankrd1* | Ankyrin Repeat Domain 1 | ^74, 75^ |
| *Cd247* | CD247 Molecule | ^76^ |
| *Hbegf* | Heparin Binding EGF Like Growth Factor | ^77^ |
| *Vegfc* | Vascular Endothelial Growth Factor C | ^70, 78^ |
| *Nos2* | Nitric Oxide Synthase 2 | ^79^ |
| *Nrarp* | NOTCH Regulated Ankyrin Repeat Protein | ^80^ |
| *Rgs2* | Regulator of G Protein Signaling 2 | ^81^ |
| *Cldn5* | Claudin 5 | ^82^ |
| *Kit* | KIT Proto-Oncogene, Receptor Tyrosine Kinase | ^83^ |
| *Cd200* | CD200 Molecule | ^84^ |
| *Gja4* | Gap Junction Protein Alpha 4 | ^85, 86^ |
| *Nog* | Noggin | ^87, 88^ |
| *Ets1* | Ets Proto-Oncogene 1, Transcription factor | ^89, 90^ |
| *Hes1* | Hes Family BHLH Transcription Factor 1 | ^91^ |

**Figure 3 – Source data 1:** Reference list for endothelial and mesenchymal genes indicated in the Figure 3D (HFD vs. Chow) heat map.

| Gene | Description | Reference # |
| --- | --- | --- |
| *Apln* | Apelin | ^15^ |
| *Vwa1* | Von Willebrand Factor A Domain Containing 1 | ^23, 24^ |
| *Tuba1a* | Tubulin Alpha 1a | ^31^ |
| *Ezh2* | Enhancer Of Zeste 2 Polycomb Repressive Complex 2 Subunit | ^74^ |
| *Fsd2* | Fibronectin Type III And SPRY Domain Containing 2 | ^92-94^ |
| *Mest* | Mesoderm Specific Transcript | ^33^ |
| *Angptl4* | Angiopoietin Like 4 | ^95^ |
| *Me1* | Malic Enzyme 1 | ^96^ |
| *Cldn5* | Claudin 5 | ^82^ |
| *Ankrd1* | Ankyrin Repeat Domain 1 | ^74, 75^ |
| *E2f1* | E2F Transcription Factor 1 | ^97^ |
| *Hey1* | Hes Related Family BHLH Transcription Factor With YRPW Motif 1 | ^52, 98^ |
| *Fndc5* | Fibronectin Type III Domain Containing 5 | ^99^ |
| *Dsp* | Desmoplakin | ^100^ |
| *Trp53inp2* | Tumor Protein P53 Inducible Nuclear Protein 2 | ^101^ |
| *Cd36* | CD36 Molecule | ^102^ |
| *Hist4h4* | Histone H4 | ^74, 98^ |
| *Xbp1* | X-Box Binding Protein 1 | ^103^ |
| *Inhbb* | Inhibin Subunit Beta B | ^104^ |
| *Podxl* | Podocalyxin Like | ^105^ |
| *Egr1* | Early Growth Response 1 | ^106^ |
| *L1cam* | L1 Cell Adhesion Molecule | ^107^ |
| *Rarg* | Retinoic Acid Receptor Gamma | ^108^ |
| *Ace* | Angiotensin I Converting Enzyme | ^109^ |
| *Apoe* | Apolipoprotein E | ^110^ |
| *Mkl2* | Myocardin-like protein 2 | ^111^ |
| *Tln2* | Talin 2 | ^112^ |
| *Pdgfrb* | Platelet Derived Growth Factor Receptor Beta | ^113^ |
| *Igfbp3* | Insulin Like Growth Factor Binding Protein 3 | ^114^ |

**Figure 3 – Source data 1:** Reference list for endothelial and mesenchymal genes indicated in the Figure 3E (TAC (2) vs. Sham) heat map.

| Gene | Description | Reference # |
| --- | --- | --- |
| *Spry4* | Sprouty RTK Signalling Antagonists | ^115^ |
| *Mmp14* | Matrix Metallopeptidase 14 | ^116^ |
| *Tnc* | Tenascin C | ^117, 118^ |
| *Fn1* | Fibronectin 1 | ^119^ |
| *Vim* | Vimentin | ^120^ |
| *Emilin1* | Elastin Microfibril Interfacer 1 | ^121, 122^ |
| *Col4a1* | Collagen Type IV Alpha 1 Chain | ^28^ |
| *Tgfb1* | Transforming Growth Factor Beta 1 | ^123, 124^ |
| *Tgfbr2* | Transforming Growth Factor Beta Receptor 2 | ^123, 124^ |
| *Ltbp3* | Latent Transforming Growth Factor Beta Binding Protein 3 | ^40^ |
| *Bmp2* | Bone Morphogenetic Protein 2 | ^125^ |
| *Lamb1* | Laminin Subunit Beta 1 | ^39^ |
| *Msn* | Moesin | ^47-49^ |
| *Sele* | Selectin E | ^126^ |
| *Acta1* | Alpha-Actin-1 | ^127^ |
| *Tagln2* | Transgelin 2 | ^45^ |
| *Hif1a* | Hypoxia Inducible Factor 1 Subunit Alpha | ^29^ |
| *Adamts4* | ADAM Metallopeptidase With Thrombospondin Type 1 Motif 4 | ^25^ |
| *Nrp2* | Neuropilin 2 | ^128^ |
| *Esm1* | Endothelial Cell Specific Molecule 1 | ^129-131^ |
| *Apln* | Apelin | ^15^ |
| *Cd34* | CD34 Molecule | ^132, 133^ |
| *Vash1* | Vasohibin 1 | ^17, 18^ |
| *Vwa1* | Von Willebrand Factor A Domain Containing 1 | ^23, 24^ |
| *Epas1* | Endothelial PAS Domain Protein 1 | ^134^ |
| *Apoe* | Apolipoprotein E | ^110^ |
| *Angpt2* | Angiopoietin | ^135^ |
| *Id2* | Inhibitor of DNA Binding 2 | ^136^ |
| *Id3* | Inhibitor of DNA Binding 3, HLH protein | ^136^ |
| *Efnb1* | Ephrin B1 | ^137^ |
| *Igf1r* | Insulin Like Growth Factor 1 Receptor | ^138^ |

**Figure 3 – Source data 1:** Reference list for endothelial and mesenchymal genes indicated in the Figure F (TAC (7) vs. Sham) heat map.

| Gene | Description | Reference  # |
| --- | --- | --- |
| *Spry4* | Sprouty RTK Signaling Antagonist 4 | ^115^ |
| *Esm1* | Endothelial Cell Specific Molecule 1 | ^129-131^ |
| *Epha2* | EPH Receptor A2 | ^137^ |
| *Socs5* | Suppressor Of Cytokine Signaling 5 | ^50^ |
| *Sele* | Selectin E | ^126^ |
| *Acta1* | Alpha-Actin-1 | ^127^ |
| *Nostrin* | Nitric Oxide Synthase Trafficking | ^139^ |
| *Esam* | Endothelial Cell Adhesion Molecule | ^140^ |
| *Cd34* | CD34 molecule | ^132, 133^ |
| *Tgfb1* | Transforming Growth Factor Beta 1 | ^123, 124^ |
| *Tnfaip2* | TNF Alpha Induced Protein 2 | ^141^ |
| *Ankrd1* | Ankyrin Repeat Domain 1 | ^25,26^ |
| *Mmp14* | Matrix Metallopeptidase 14 | ^116^ |
| *Vwf* | Von Willebrand Factor | ^142^ |
| *Col4a1* | Collagen Type IV Alpha 1 Chain | ^27, 28^ |
| *Tnc* | Tenascin C | ^117, 118^ |
| *Col4a2* | Collagen Type IV Alpha 2 Chain | ^27, 28^ |
| *Stat3* | Signal Transducer And Activator Of Transcription 3 | ^143^ |
| *Ecm1* | Extracellular Matrix Protein 1 | ^144^ |
| *Zeb1* | Zinc Finger E-Box Binding Homeobox 1 | ^143^ |
| *Emilin1* | Elastin Microfibril Interfacer 1 | ^121, 122^ |
| *Sparc* | Secreted Protein Acidic And Cysteine Rich | ^26^ |
| *Dsp* | Desmoplakin | ^100^ |
| *Notch3* | NOTCH Receptor 3 | ^113, 144^ |
| *Pdgfrb* | Platelet Derived Growth Factor Receptor Beta | ^113^ |
| *Epha4* | EPH Receptor 4 | ^137^ |
| *Ddr2* | Discoidin Domain Receptor Tyrosine Kinase 2 | ^145^ |
| *Zeb2* | Zinc Finger E-Box Binding Homeobox 2 | ^70^ |
| *Apoe* | Apolipoprotein E | ^110^ |
| *Sox7* | SRY-Box Transcription Factor 7 | ^146^ |
| *Cxcl9* | C-X-C Motif Chemokine Ligand 9 | ^147^ |

**References**

1.Michalik KM, You X, Manavski Y, Doddaballapur A, Zornig M, Braun T, John D, Ponomareva Y, Chen W, Uchida S, Boon RA and Dimmeler S. Long noncoding RNA MALAT1 regulates endothelial cell function and vessel growth. *Circ Res*. 2014;114:1389-97.

2.Bostrom K, Zebboudj AF, Yao Y, Lin TS and Torres A. Matrix GLA protein stimulates VEGF expression through increased transforming growth factor-beta1 activity in endothelial cells. *J Biol Chem*. 2004;279:52904-13.

3.Neilsen PM, Cheney KM, Li CW, Chen JD, Cawrse JE, Schulz RB, Powell JA, Kumar R and Callen DF. Identification of ANKRD11 as a p53 coactivator. *J Cell Sci*. 2008;121:3541-52.

4.Rhee SG and Bae YS. Regulation of phosphoinositide-specific phospholipase C isozymes. *J Biol Chem*. 1997;272:15045-8.

5.Lisowska J, Rodel CJ, Manet S, Miroshnikova YA, Boyault C, Planus E, De Mets R, Lee HH, Destaing O, Mertani H, Boulday G, Tournier-Lasserve E, Balland M, Abdelilah-Seyfried S, Albiges-Rizo C and Faurobert E. The CCM1-CCM2 complex controls complementary functions of ROCK1 and ROCK2 that are required for endothelial integrity. *J Cell Sci*. 2018;131.

6.Wu S, Ou T, Xing N, Lu J, Wan S, Wang C, Zhang X, Yang F, Huang Y and Cai Z. Whole-genome sequencing identifies ADGRG6 enhancer mutations and FRS2 duplications as angiogenesis-related drivers in bladder cancer. *Nat Commun*. 2019;10:720.

7.Kim BR, Seo SH, Park MS, Lee SH, Kwon Y and Rho SB. sMEK1 inhibits endothelial cell proliferation by attenuating VEGFR-2-dependent-Akt/eNOS/HIF-1alpha signaling pathways. *Oncotarget*. 2015;6:31830-43.

8.Farhang-Fallah J, Yin X, Trentin G, Cheng AM and Rozakis-Adcock M. Cloning and characterization of PHIP, a novel insulin receptor substrate-1 pleckstrin homology domain interacting protein. *J Biol Chem*. 2000;275:40492-7.

9.Bowler E, Porazinski S, Uzor S, Thibault P, Durand M, Lapointe E, Rouschop KMA, Hancock J, Wilson I and Ladomery M. Hypoxia leads to significant changes in alternative splicing and elevated expression of CLK splice factor kinases in PC3 prostate cancer cells. *BMC Cancer*. 2018;18:355.

10.Bai R, Li D, Shi Z, Fang X, Ge W and Zheng S. Clinical significance of Ankyrin repeat domain 12 expression in colorectal cancer. *J Exp Clin Cancer Res*. 2013;32:35.

11.Besnard V, Dagher R, Madjer T, Joannes A, Jaillet M, Kolb M, Bonniaud P, Murray LA, Sleeman MA and Crestani B. Identification of periplakin as a major regulator of lung injury and repair in mice. *JCI Insight*. 2018;3.

12.Zhou Z, Tang AT, Wong WY, Bamezai S, Goddard LM, Shenkar R, Zhou S, Yang J, Wright AC, Foley M, Arthur JS, Whitehead KJ, Awad IA, Li DY, Zheng X and Kahn ML. Cerebral cavernous malformations arise from endothelial gain of MEKK3-KLF2/4 signalling. *Nature*. 2016;532:122-6.

13.Bravi L, Malinverno M, Pisati F, Rudini N, Cuttano R, Pallini R, Martini M, Larocca LM, Locatelli M, Levi V, Bertani GA, Dejana E and Lampugnani MG. Endothelial Cells Lining Sporadic Cerebral Cavernous Malformation Cavernomas Undergo Endothelial-to-Mesenchymal Transition. *Stroke*. 2016;47:886-90.

14.Dackor RT, Fritz-Six K, Dunworth WP, Gibbons CL, Smithies O and Caron KM. Hydrops fetalis, cardiovascular defects, and embryonic lethality in mice lacking the calcitonin receptor-like receptor gene. *Mol Cell Biol*. 2006;26:2511-8.

15.Wysocka MB, Pietraszek-Gremplewicz K and Nowak D. The Role of Apelin in Cardiovascular Diseases, Obesity and Cancer. *Front Physiol*. 2018;9:557.

16.Apostolidis SA, Stifano G, Tabib T, Rice LM, Morse CM, Kahaleh B and Lafyatis R. Single Cell RNA Sequencing Identifies HSPG2 and APLNR as Markers of Endothelial Cell Injury in Systemic Sclerosis Skin. *Front Immunol*. 2018;9:2191.

17.Watanabe K, Hasegawa Y, Yamashita H, Shimizu K, Ding Y, Abe M, Ohta H, Imagawa K, Hojo K, Maki H, Sonoda H and Sato Y. Vasohibin as an endothelium-derived negative feedback regulator of angiogenesis. *J Clin Invest*. 2004;114:898-907.

18.Sato Y. The vasohibin family: a novel family for angiogenesis regulation. *J Biochem*. 2013;153:5-11.

19.Wang CQ, Tang CH, Wang Y, Jin L, Wang Q, Li X, Hu GN, Huang BF, Zhao YM and Su CM. FSCN1 gene polymorphisms: biomarkers for the development and progression of breast cancer. *Sci Rep*. 2017;7:15887.

20.Galvagni F, Nardi F, Maida M, Bernardini G, Vannuccini S, Petraglia F, Santucci A and Orlandini M. CD93 and dystroglycan cooperation in human endothelial cell adhesion and migration adhesion and migration. *Oncotarget*. 2016;7:10090-103.

21.Lugano R, Vemuri K, Yu D, Bergqvist M, Smits A, Essand M, Johansson S, Dejana E and Dimberg A. CD93 promotes beta1 integrin activation and fibronectin fibrillogenesis during tumor angiogenesis. *J Clin Invest*. 2018;128:3280-3297.

22.Fitzgerald J. WARP: A Unique Extracellular Matrix Component of Cartilage, Muscle, and Endothelial Cell Basement Membranes. *Anat Rec (Hoboken)*. 2019.

23.Deckx S, Carai P, Bateman J, Heymans S and Papageorgiou AP. Breeding Strategy Determines Rupture Incidence in Post-Infarct Healing WARPing Cardiovascular Research. *PLoS One*. 2015;10:e0139199.

24.Hsu YP, Staton CA, Cross N and Buttle DJ. Anti-angiogenic properties of ADAMTS-4 in vitro. *Int J Exp Pathol*. 2012;93:70-7.

25.Fenouille N, Tichet M, Dufies M, Pottier A, Mogha A, Soo JK, Rocchi S, Mallavialle A, Galibert MD, Khammari A, Lacour JP, Ballotti R, Deckert M and Tartare-Deckert S. The epithelial-mesenchymal transition (EMT) regulatory factor SLUG (SNAI2) is a downstream target of SPARC and AKT in promoting melanoma cell invasion. *PLoS One*. 2012;7:e40378.

26.Katsuda S, Okada Y, Minamoto T, Oda Y, Matsui Y and Nakanishi I. Collagens in human atherosclerosis. Immunohistochemical analysis using collagen type-specific antibodies. *Arterioscler Thromb*. 1992;12:494-502.

27.Yang W, Ng FL, Chan K, Pu X, Poston RN, Ren M, An W, Zhang R, Wu J, Yan S, Situ H, He X, Chen Y, Tan X, Xiao Q, Tucker AT, Caulfield MJ and Ye S. Coronary-Heart-Disease-Associated Genetic Variant at the COL4A1/COL4A2 Locus Affects COL4A1/COL4A2 Expression, Vascular Cell Survival, Atherosclerotic Plaque Stability and Risk of Myocardial Infarction. *PLoS Genet*. 2016;12:e1006127.

28.Sanchez-Duffhues G, Garcia de Vinuesa A and Ten Dijke P. Endothelial-to-mesenchymal transition in cardiovascular diseases: Developmental signaling pathways gone awry. *Dev Dyn*. 2018;247:492-508.

29.Kitao A, Sato Y, Sawada-Kitamura S, Harada K, Sasaki M, Morikawa H, Shiomi S, Honda M, Matsui O and Nakanuma Y. Endothelial to mesenchymal transition via transforming growth factor-beta1/Smad activation is associated with portal venous stenosis in idiopathic portal hypertension. *Am J Pathol*. 2009;175:616-26.

30.Groger CJ, Grubinger M, Waldhor T, Vierlinger K and Mikulits W. Meta-analysis of gene expression signatures defining the epithelial to mesenchymal transition during cancer progression. *PLoS One*. 2012;7:e51136.

31.Imaizumi T, Yoshida H and Satoh K. Regulation of CX3CL1/fractalkine expression in endothelial cells. *J Atheroscler Thromb*. 2004;11:15-21.

32.Mayer W, Hemberger M, Frank HG, Grummer R, Winterhager E, Kaufmann P and Fundele R. Expression of the imprinted genes MEST/Mest in human and murine placenta suggests a role in angiogenesis. *Dev Dyn*. 2000;217:1-10.

33.Liu R and Jin JP. Calponin isoforms CNN1, CNN2 and CNN3: Regulators for actin cytoskeleton functions in smooth muscle and non-muscle cells. *Gene*. 2016;585:143-153.

34.Cho SH, Park YS, Kim HJ, Kim CH, Lim SW, Huh JW, Lee JH and Kim HR. CD44 enhances the epithelial-mesenchymal transition in association with colon cancer invasion. *Int J Oncol*. 2012;41:211-8.

35.Choi SH, Kim AR, Nam JK, Kim JM, Kim JY, Seo HR, Lee HJ, Cho J and Lee YJ. Tumour-vasculature development via endothelial-to-mesenchymal transition after radiotherapy controls CD44v6(+) cancer cell and macrophage polarization. *Nat Commun*. 2018;9:5108.

36.Naikawadi RP, Cheng N, Vogel SM, Qian F, Wu D, Malik AB and Ye RD. A critical role for phosphatidylinositol (3,4,5)-trisphosphate-dependent Rac exchanger 1 in endothelial junction disruption and vascular hyperpermeability. *Circ Res*. 2012;111:1517-27.

37.Wolf FW, Marks RM, Sarma V, Byers MG, Katz RW, Shows TB and Dixit VM. Characterization of a novel tumor necrosis factor-alpha-induced endothelial primary response gene. *J Biol Chem*. 1992;267:1317-26.

38.Wagner JUG, Chavakis E, Rogg EM, Muhly-Reinholz M, Glaser SF, Gunther S, John D, Bonini F, Zeiher AM, Schaefer L, Hannocks MJ, Boon RA and Dimmeler S. Switch in Laminin beta2 to Laminin beta1 Isoforms During Aging Controls Endothelial Cell Functions-Brief Report. *Arterioscler Thromb Vasc Biol*. 2018;38:1170-1177.

39.Goumans MJ and Ten Dijke P. TGF-beta Signaling in Control of Cardiovascular Function. *Cold Spring Harb Perspect Biol*. 2018;10.

40.Neubauer K, Neubauer B, Seidl M and Zieger B. Characterization of septin expression in normal and fibrotic kidneys. *Cytoskeleton (Hoboken)*. 2019;76:143-153.

41.Lu X, Le Noble F, Yuan L, Jiang Q, De Lafarge B, Sugiyama D, Breant C, Claes F, De Smet F, Thomas JL, Autiero M, Carmeliet P, Tessier-Lavigne M and Eichmann A. The netrin receptor UNC5B mediates guidance events controlling morphogenesis of the vascular system. *Nature*. 2004;432:179-86.

42.Tang J, Zhang H, He L, Huang X, Li Y, Pu W, Yu W, Zhang L, Cai D, Lui KO and Zhou B. Genetic Fate Mapping Defines the Vascular Potential of Endocardial Cells in the Adult Heart. *Circ Res*. 2018;122:984-993.

43.Zhang H, Huang X, Liu K, Tang J, He L, Pu W, Liu Q, Li Y, Tian X, Wang Y, Zhang L, Yu Y, Wang H, Hu R, Wang F, Chen T, Wang QD, Qiao Z, Zhang L, Lui KO and Zhou B. Fibroblasts in an endocardial fibroelastosis disease model mainly originate from mesenchymal derivatives of epicardium. *Cell Res*. 2017;27:1157-1177.

44.Xiao Y, Li Y, Han J, Pan Y, Tie L and Li X. Transgelin 2 participates in lovastatin-induced anti-angiogenic effects in endothelial cells through a phosphorylated myosin light chain-related mechanism. *PLoS One*. 2012;7:e46510.

45.Piera-Velazquez S and Jimenez SA. Endothelial to Mesenchymal Transition: Role in Physiology and in the Pathogenesis of Human Diseases. *Physiol Rev*. 2019;99:1281-1324.

46.Adyshev DM, Dudek SM, Moldobaeva N, Kim KM, Ma SF, Kasa A, Garcia JG and Verin AD. Ezrin/radixin/moesin proteins differentially regulate endothelial hyperpermeability after thrombin. *Am J Physiol Lung Cell Mol Physiol*. 2013;305:L240-55.

47.Haynes J, Srivastava J, Madson N, Wittmann T and Barber DL. Dynamic actin remodeling during epithelial-mesenchymal transition depends on increased moesin expression. *Mol Biol Cell*. 2011;22:4750-64.

48.Lamouille S, Xu J and Derynck R. Molecular mechanisms of epithelial-mesenchymal transition. *Nat Rev Mol Cell Biol*. 2014;15:178-96.

49.Linossi EM, Chandrashekaran IR, Kolesnik TB, Murphy JM, Webb AI, Willson TA, Kedzierski L, Bullock AN, Babon JJ, Norton RS, Nicola NA and Nicholson SE. Suppressor of Cytokine Signaling (SOCS) 5 utilises distinct domains for regulation of JAK1 and interaction with the adaptor protein Shc-1. *PLoS One*. 2013;8:e70536.

50.Yousef H, Czupalla CJ, Lee D, Chen MB, Burke AN, Zera KA, Zandstra J, Berber E, Lehallier B, Mathur V, Nair RV, Bonanno LN, Yang AC, Peterson T, Hadeiba H, Merkel T, Korbelin J, Schwaninger M, Buckwalter MS, Quake SR, Butcher EC and Wyss-Coray T. Aged blood impairs hippocampal neural precursor activity and activates microglia via brain endothelial cell VCAM1. *Nat Med*. 2019;25:988-1000.

51.Niessen K, Fu Y, Chang L, Hoodless PA, McFadden D and Karsan A. Slug is a direct Notch target required for initiation of cardiac cushion cellularization. *J Cell Biol*. 2008;182:315-25.

52.Nishimura Y, Nitto T, Inoue T and Node K. STAT6 mediates apoptosis of human coronary arterial endothelial cells by interleukin-13. *Hypertens Res*. 2008;31:535-41.

53.Hubmacher D and Apte SS. ADAMTS proteins as modulators of microfibril formation and function. *Matrix Biol*. 2015;47:34-43.

54.Serbanovic-Canic J, de Luca A, Warboys C, Ferreira PF, Luong LA, Hsiao S, Gauci I, Mahmoud M, Feng S, Souilhol C, Bowden N, Ashton JP, Walczak H, Firmin D, Krams R, Mason JC, Haskard DO, Sherwin S, Ridger V, Chico TJ and Evans PC. Zebrafish Model for Functional Screening of Flow-Responsive Genes. *Arterioscler Thromb Vasc Biol*. 2017;37:130-143.

55.Souilhol C, Serbanovic-Canic J, Fragiadaki M, Chico TJ, Ridger V, Roddie H and Evans PC. Endothelial responses to shear stress in atherosclerosis: a novel role for developmental genes. *Nat Rev Cardiol*. 2020;17:52-63.

56.Xiang W, Ke Z, Zhang Y, Cheng GH, Irwan ID, Sulochana KN, Potturi P, Wang Z, Yang H, Wang J, Zhuo L, Kini RM and Ge R. Isthmin is a novel secreted angiogenesis inhibitor that inhibits tumour growth in mice. *J Cell Mol Med*. 2011;15:359-74.

57.Venugopal S, Chen M, Liao W, Er SY, Wong WS and Ge R. Isthmin is a novel vascular permeability inducer that functions through cell-surface GRP78-mediated Src activation. *Cardiovasc Res*. 2015;107:131-42.

58.Mesbah K, Harrelson Z, Theveniau-Ruissy M, Papaioannou VE and Kelly RG. Tbx3 is required for outflow tract development. *Circ Res*. 2008;103:743-50.

59.Valverius EM, Bates SE, Stampfer MR, Clark R, McCormick F, Salomon DS, Lippman ME and Dickson RB. Transforming growth factor alpha production and epidermal growth factor receptor expression in normal and oncogene transformed human mammary epithelial cells. *Mol Endocrinol*. 1989;3:203-14.

60.Yakala GK, Cabrera-Fuentes HA, Crespo-Avilan GE, Rattanasopa C, Burlacu A, George BL, Anand K, Mayan DC, Corliano M, Hernandez-Resendiz S, Wu Z, Schwerk AMK, Tan ALJ, Trigueros-Motos L, Chevre R, Chua T, Kleemann R, Liehn EA, Hausenloy DJ, Ghosh S and Singaraja RR. FURIN Inhibition Reduces Vascular Remodeling and Atherosclerotic Lesion Progression in Mice. *Arterioscler Thromb Vasc Biol*. 2019;39:387-401.

61.ten Dijke P, Goumans MJ and Pardali E. Endoglin in angiogenesis and vascular diseases. *Angiogenesis*. 2008;11:79-89.

62.van Meeteren LA and ten Dijke P. Regulation of endothelial cell plasticity by TGF-beta. *Cell Tissue Res*. 2012;347:177-86.

63.Topper JN, Cai J, Qiu Y, Anderson KR, Xu YY, Deeds JD, Feeley R, Gimeno CJ, Woolf EA, Tayber O, Mays GG, Sampson BA, Schoen FJ, Gimbrone MA, Jr. and Falb D. Vascular MADs: two novel MAD-related genes selectively inducible by flow in human vascular endothelium. *Proc Natl Acad Sci U S A*. 1997;94:9314-9.

64.Lebrin F, Deckers M, Bertolino P and Ten Dijke P. TGF-beta receptor function in the endothelium. *Cardiovasc Res*. 2005;65:599-608.

65.Goveia J, Zecchin A, Rodriguez FM, Moens S, Stapor P and Carmeliet P. Endothelial cell differentiation by SOX17: promoting the tip cell or stalking its neighbor instead? *Circ Res*. 2014;115:205-7.

66.Zhang H, Liu CY, Zha ZY, Zhao B, Yao J, Zhao S, Xiong Y, Lei QY and Guan KL. TEAD transcription factors mediate the function of TAZ in cell growth and epithelial-mesenchymal transition. *J Biol Chem*. 2009;284:13355-62.

67.Dunn J, Qiu H, Kim S, Jjingo D, Hoffman R, Kim CW, Jang I, Son DJ, Kim D, Pan C, Fan Y, Jordan IK and Jo H. Flow-dependent epigenetic DNA methylation regulates endothelial gene expression and atherosclerosis. *J Clin Invest*. 2014;124:3187-99.

68.Sweet DR, Fan L, Hsieh PN and Jain MK. Kruppel-Like Factors in Vascular Inflammation: Mechanistic Insights and Therapeutic Potential. *Front Cardiovasc Med*. 2018;5:6.

69.Kovacic JC, Dimmeler S, Harvey RP, Finkel T, Aikawa E, Krenning G and Baker AH. Endothelial to Mesenchymal Transition in Cardiovascular Disease: JACC State-of-the-Art Review. *J Am Coll Cardiol*. 2019;73:190-209.

70.Glaser SF, Heumuller AW, Tombor L, Hofmann P, Muhly-Reinholz M, Fischer A, Gunther S, Kokot KE, Hassel D, Kumar S, Jo H, Boon RA, Abplanalp W, John D, Boeckel JN and Dimmeler S. The histone demethylase JMJD2B regulates endothelial-to-mesenchymal transition. *Proc Natl Acad Sci U S A*. 2020;117:4180-4187.

71.von Gise A, Zhou B, Honor LB, Ma Q, Petryk A and Pu WT. WT1 regulates epicardial epithelial to mesenchymal transition through beta-catenin and retinoic acid signaling pathways. *Dev Biol*. 2011;356:421-31.

72.Kovacic JC, Mercader N, Torres M, Boehm M and Fuster V. Epithelial-to-mesenchymal and endothelial-to-mesenchymal transition: from cardiovascular development to disease. *Circulation*. 2012;125:1795-808.

73.Hulshoff MS, Xu X, Krenning G and Zeisberg EM. Epigenetic Regulation of Endothelial-to-Mesenchymal Transition in Chronic Heart Disease. *Arterioscler Thromb Vasc Biol*. 2018;38:1986-1996.

74.Kanai H, Tanaka T, Aihara Y, Takeda S, Kawabata M, Miyazono K, Nagai R and Kurabayashi M. Transforming growth factor-beta/Smads signaling induces transcription of the cell type-restricted ankyrin repeat protein CARP gene through CAGA motif in vascular smooth muscle cells. *Circ Res*. 2001;88:30-6.

75.Bruno A, Mortara L, Baci D, Noonan DM and Albini A. Myeloid Derived Suppressor Cells Interactions With Natural Killer Cells and Pro-angiogenic Activities: Roles in Tumor Progression. *Front Immunol*. 2019;10:771.

76.Ongusaha PP, Kwak JC, Zwible AJ, Macip S, Higashiyama S, Taniguchi N, Fang L and Lee SW. HB-EGF is a potent inducer of tumor growth and angiogenesis. *Cancer Res*. 2004;64:5283-90.

77.Chen HI, Sharma B, Akerberg BN, Numi HJ, Kivela R, Saharinen P, Aghajanian H, McKay AS, Bogard PE, Chang AH, Jacobs AH, Epstein JA, Stankunas K, Alitalo K and Red-Horse K. The sinus venosus contributes to coronary vasculature through VEGFC-stimulated angiogenesis. *Development*. 2014;141:4500-12.

78.Hemmrich K, Suschek CV, Lerzynski G and Kolb-Bachofen V. iNOS activity is essential for endothelial stress gene expression protecting against oxidative damage. *J Appl Physiol (1985)*. 2003;95:1937-46.

79.Phng LK, Potente M, Leslie JD, Babbage J, Nyqvist D, Lobov I, Ondr JK, Rao S, Lang RA, Thurston G and Gerhardt H. Nrarp coordinates endothelial Notch and Wnt signaling to control vessel density in angiogenesis. *Dev Cell*. 2009;16:70-82.

80.Osei-Owusu P, Sabharwal R, Kaltenbronn KM, Rhee MH, Chapleau MW, Dietrich HH and Blumer KJ. Regulator of G protein signaling 2 deficiency causes endothelial dysfunction and impaired endothelium-derived hyperpolarizing factor-mediated relaxation by dysregulating Gi/o signaling. *J Biol Chem*. 2012;287:12541-9.

81.Nitta T, Hata M, Gotoh S, Seo Y, Sasaki H, Hashimoto N, Furuse M and Tsukita S. Size-selective loosening of the blood-brain barrier in claudin-5-deficient mice. *J Cell Biol*. 2003;161:653-60.

82.Puputti M, Tynninen O, Pernila P, Salmi M, Jalkanen S, Paetau A, Sihto H and Joensuu H. Expression of KIT receptor tyrosine kinase in endothelial cells of juvenile brain tumors. *Brain Pathol*. 2010;20:763-70.

83.Ko YC, Chien HF, Jiang-Shieh YF, Chang CY, Pai MH, Huang JP, Chen HM and Wu CH. Endothelial CD200 is heterogeneously distributed, regulated and involved in immune cell-endothelium interactions. *J Anat*. 2009;214:183-95.

84.Kanady JD, Dellinger MT, Munger SJ, Witte MH and Simon AM. Connexin37 and Connexin43 deficiencies in mice disrupt lymphatic valve development and result in lymphatic disorders including lymphedema and chylothorax. *Dev Biol*. 2011;354:253-66.

85.Fang JS, Coon BG, Gillis N, Chen Z, Qiu J, Chittenden TW, Burt JM, Schwartz MA and Hirschi KK. Shear-induced Notch-Cx37-p27 axis arrests endothelial cell cycle to enable arterial specification. *Nat Commun*. 2017;8:2149.

86.Ramasamy SK, Kusumbe AP, Wang L and Adams RH. Endothelial Notch activity promotes angiogenesis and osteogenesis in bone. *Nature*. 2014;507:376-380.

87.Kang HW, Walvick R and Bogdanov A, Jr. In vitro and In vivo imaging of antivasculogenesis induced by Noggin protein expression in human venous endothelial cells. *FASEB J*. 2009;23:4126-34.

88.Oda N, Abe M and Sato Y. ETS-1 converts endothelial cells to the angiogenic phenotype by inducing the expression of matrix metalloproteinases and integrin beta3. *J Cell Physiol*. 1999;178:121-32.

89.Wei G, Srinivasan R, Cantemir-Stone CZ, Sharma SM, Santhanam R, Weinstein M, Muthusamy N, Man AK, Oshima RG, Leone G and Ostrowski MC. Ets1 and Ets2 are required for endothelial cell survival during embryonic angiogenesis. *Blood*. 2009;114:1123-30.

90.Kitagawa M, Hojo M, Imayoshi I, Goto M, Ando M, Ohtsuka T, Kageyama R and Miyamoto S. Hes1 and Hes5 regulate vascular remodeling and arterial specification of endothelial cells in brain vascular development. *Mech Dev*. 2013;130:458-66.

91.Hynes RO and Yamada KM. Fibronectins: multifunctional modular glycoproteins. *J Cell Biol*. 1982;95:369-77.

92.Bork P and Doolittle RF. Proposed acquisition of an animal protein domain by bacteria. *Proc Natl Acad Sci U S A*. 1992;89:8990-4.

93.Ponting C, Schultz J and Bork P. SPRY domains in ryanodine receptors (Ca(2+)-release channels). *Trends Biochem Sci*. 1997;22:193-4.

94.Cazes A, Galaup A, Chomel C, Bignon M, Brechot N, Le Jan S, Weber H, Corvol P, Muller L, Germain S and Monnot C. Extracellular matrix-bound angiopoietin-like 4 inhibits endothelial cell adhesion, migration, and sprouting and alters actin cytoskeleton. *Circ Res*. 2006;99:1207-15.

95.Wen D, Liu D, Tang J, Dong L, Liu Y, Tao Z, Wan J, Gao D, Wang L, Sun H, Fan J and Wu W. Malic enzyme 1 induces epithelial-mesenchymal transition and indicates poor prognosis in hepatocellular carcinoma. *Tumour Biol*. 2015;36:6211-21.

96.Wang T, Chen X, Qiao W, Kong L, Sun D and Li Z. Transcription factor E2F1 promotes EMT by regulating ZEB2 in small cell lung cancer. *BMC Cancer*. 2017;17:719.

97.Fu Y, Chang A, Chang L, Niessen K, Eapen S, Setiadi A and Karsan A. Differential regulation of transforming growth factor beta signaling pathways by Notch in human endothelial cells. *J Biol Chem*. 2009;284:19452-62.

98.Bian T, Zheng L, Jiang D, Liu J, Zhang J, Feng J, Zhang Q, Qian L, Qiu H, Liu Y and Yao S. Overexpression of fibronectin type III domain containing 3B is correlated with epithelial-mesenchymal transition and predicts poor prognosis in lung adenocarcinoma. *Exp Ther Med*. 2019;17:3317-3326.

99.Delmar M and McKenna WJ. The cardiac desmosome and arrhythmogenic cardiomyopathies: from gene to disease. *Circ Res*. 2010;107:700-14.

100.Shahbazi J, Lock R and Liu T. Tumor Protein 53-Induced Nuclear Protein 1 Enhances p53 Function and Represses Tumorigenesis. *Front Genet*. 2013;4:80.

101.Helmke A, Casper J, Nordlohne J, David S, Haller H, Zeisberg EM and von Vietinghoff S. Endothelial-to-mesenchymal transition shapes the atherosclerotic plaque and modulates macrophage function. *FASEB J*. 2019;33:2278-2289.

102.Zeng L, Zampetaki A, Margariti A, Pepe AE, Alam S, Martin D, Xiao Q, Wang W, Jin ZG, Cockerill G, Mori K, Li YS, Hu Y, Chien S and Xu Q. Sustained activation of XBP1 splicing leads to endothelial apoptosis and atherosclerosis development in response to disturbed flow. *Proc Natl Acad Sci U S A*. 2009;106:8326-31.

103.Myllarniemi M, Tikkanen J, Hulmi JJ, Pasternack A, Sutinen E, Ronty M, Lepparanta O, Ma H, Ritvos O and Koli K. Upregulation of activin-B and follistatin in pulmonary fibrosis - a translational study using human biopsies and a specific inhibitor in mouse fibrosis models. *BMC Pulm Med*. 2014;14:170.

104.Horrillo A, Porras G, Ayuso MS and Gonzalez-Manchon C. Loss of endothelial barrier integrity in mice with conditional ablation of podocalyxin (Podxl) in endothelial cells. *Eur J Cell Biol*. 2016;95:265-76.

105.Abdel-Malak NA, Mofarrahi M, Mayaki D, Khachigian LM and Hussain SN. Early growth response-1 regulates angiopoietin-1-induced endothelial cell proliferation, migration, and differentiation. *Arterioscler Thromb Vasc Biol*. 2009;29:209-16.

106.Magrini E, Villa A, Angiolini F, Doni A, Mazzarol G, Rudini N, Maddaluno L, Komuta M, Topal B, Prenen H, Schachner M, Confalonieri S, Dejana E, Bianchi F, Mazzone M and Cavallaro U. Endothelial deficiency of L1 reduces tumor angiogenesis and promotes vessel normalization. *J Clin Invest*. 2014;124:4335-50.

107.Mizee MR, Wooldrik D, Lakeman KA, van het Hof B, Drexhage JA, Geerts D, Bugiani M, Aronica E, Mebius RE, Prat A, de Vries HE and Reijerkerk A. Retinoic acid induces blood-brain barrier development. *J Neurosci*. 2013;33:1660-71.

108.Weckler N, Leitzbach D, Kalinowski L, Malinski T, Busch AE and Linz W. Effect of chronic treatment with the vasopeptidase inhibitor AVE 7688 and ramipril on endothelial function in atherogenic diet rabbits. *J Renin Angiotensin Aldosterone Syst*. 2003;4:191-6.

109.Bostrom KI, Yao J, Guihard PJ, Blazquez-Medela AM and Yao Y. Endothelial-mesenchymal transition in atherosclerotic lesion calcification. *Atherosclerosis*. 2016;253:124-127.

110.Li J, Bowens N, Cheng L, Zhu X, Chen M, Hannenhalli S, Cappola TP and Parmacek MS. Myocardin-like protein 2 regulates TGFbeta signaling in embryonic stem cells and the developing vasculature. *Development*. 2012;139:3531-42.

111.Monkley SJ, Kostourou V, Spence L, Petrich B, Coleman S, Ginsberg MH, Pritchard CA and Critchley DR. Endothelial cell talin1 is essential for embryonic angiogenesis. *Dev Biol*. 2011;349:494-502.

112.Battegay EJ, Rupp J, Iruela-Arispe L, Sage EH and Pech M. PDGF-BB modulates endothelial proliferation and angiogenesis in vitro via PDGF beta-receptors. *J Cell Biol*. 1994;125:917-28.

113.Granata R, Trovato L, Lupia E, Sala G, Settanni F, Camussi G, Ghidoni R and Ghigo E. Insulin-like growth factor binding protein-3 induces angiogenesis through IGF-I- and SphK1-dependent mechanisms. *J Thromb Haemost*. 2007;5:835-45.

114.Gong Y, Yang X, He Q, Gower L, Prudovsky I, Vary CP, Brooks PC and Friesel RE. Sprouty4 regulates endothelial cell migration via modulating integrin beta3 stability through c-Src. *Angiogenesis*. 2013;16:861-75.

115.Taylor SH, Yeung CY, Kalson NS, Lu Y, Zigrino P, Starborg T, Warwood S, Holmes DF, Canty-Laird EG, Mauch C and Kadler KE. Matrix metalloproteinase 14 is required for fibrous tissue expansion. *Elife*. 2015;4:e09345.

116.Bhattacharyya S, Wang W, Morales-Nebreda L, Feng G, Wu M, Zhou X, Lafyatis R, Lee J, Hinchcliff M, Feghali-Bostwick C, Lakota K, Budinger GR, Raparia K, Tamaki Z and Varga J. Tenascin-C drives persistence of organ fibrosis. *Nat Commun*. 2016;7:11703.

117.Ummarino D. Systemic sclerosis: Tenascin C perpetuates tissue fibrosis. *Nat Rev Rheumatol*. 2016;12:375.

118.Valiente-Alandi I, Potter SJ, Salvador AM, Schafer AE, Schips T, Carrillo-Salinas F, Gibson AM, Nieman ML, Perkins C, Sargent MA, Huo J, Lorenz JN, DeFalco T, Molkentin JD, Alcaide P and Blaxall BC. Inhibiting Fibronectin Attenuates Fibrosis and Improves Cardiac Function in a Model of Heart Failure. *Circulation*. 2018;138:1236-1252.

119.Wang Z, Divanyan A, Jourd'heuil FL, Goldman RD, Ridge KM, Jourd'heuil D and Lopez-Soler RI. Vimentin expression is required for the development of EMT-related renal fibrosis following unilateral ureteral obstruction in mice. *Am J Physiol Renal Physiol*. 2018;315:F769-F780.

120.Mongiat M, Ligresti G, Marastoni S, Lorenzon E, Doliana R and Colombatti A. Regulation of the extrinsic apoptotic pathway by the extracellular matrix glycoprotein EMILIN2. *Mol Cell Biol*. 2007;27:7176-87.

121.Schiavinato A, Keene DR, Imhof T, Doliana R, Sasaki T and Sengle G. Fibulin-4 deposition requires EMILIN-1 in the extracellular matrix of osteoblasts. *Sci Rep*. 2017;7:5526.

122.Cooley BC, Nevado J, Mellad J, Yang D, St Hilaire C, Negro A, Fang F, Chen G, San H, Walts AD, Schwartzbeck RL, Taylor B, Lanzer JD, Wragg A, Elagha A, Beltran LE, Berry C, Feil R, Virmani R, Ladich E, Kovacic JC and Boehm M. TGF-beta signaling mediates endothelial-to-mesenchymal transition (EndMT) during vein graft remodeling. *Sci Transl Med*. 2014;6:227ra34.

123.Man S, Sanchez Duffhues G, Ten Dijke P and Baker D. The therapeutic potential of targeting the endothelial-to-mesenchymal transition. *Angiogenesis*. 2019;22:3-13.

124.Luna-Zurita L, Prados B, Grego-Bessa J, Luxan G, del Monte G, Benguria A, Adams RH, Perez-Pomares JM and de la Pompa JL. Integration of a Notch-dependent mesenchymal gene program and Bmp2-driven cell invasiveness regulates murine cardiac valve formation. *J Clin Invest*. 2010;120:3493-507.

125.Azuma A, Takahashi S, Nose M, Araki K, Araki M, Takahashi T, Hirose M, Kawashima H, Miyasaka M and Kudoh S. Role of E-selectin in bleomycin induced lung fibrosis in mice. *Thorax*. 2000;55:147-52.

126.Kant S, Freytag B, Herzog A, Reich A, Merkel R, Hoffmann B, Krusche CA and Leube RE. Desmoglein 2 mutation provokes skeletal muscle actin expression and accumulation at intercalated discs in murine hearts. *J Cell Sci*. 2019;132.

127.Nasarre P, Gemmill RM, Potiron VA, Roche J, Lu X, Baron AE, Korch C, Garrett-Mayer E, Lagana A, Howe PH and Drabkin HA. Neuropilin-2 Is upregulated in lung cancer cells during TGF-beta1-induced epithelial-mesenchymal transition. *Cancer Res*. 2013;73:7111-21.

128.Lassalle P, Molet S, Janin A, Heyden JV, Tavernier J, Fiers W, Devos R and Tonnel AB. ESM-1 is a novel human endothelial cell-specific molecule expressed in lung and regulated by cytokines. *J Biol Chem*. 1996;271:20458-64.

129.Rocha SF, Schiller M, Jing D, Li H, Butz S, Vestweber D, Biljes D, Drexler HC, Nieminen-Kelha M, Vajkoczy P, Adams S, Benedito R and Adams RH. Esm1 modulates endothelial tip cell behavior and vascular permeability by enhancing VEGF bioavailability. *Circ Res*. 2014;115:581-90.

130.De Freitas Caires N and Lassalle P. Highlight on mouse endocan. *Circ Res*. 2015;116:e69-70.

131.Sidney LE, Branch MJ, Dunphy SE, Dua HS and Hopkinson A. Concise review: evidence for CD34 as a common marker for diverse progenitors. *Stem Cells*. 2014;32:1380-9.

132.Friedrich EB, Walenta K, Scharlau J, Nickenig G and Werner N. CD34-/CD133+/VEGFR-2+ endothelial progenitor cell subpopulation with potent vasoregenerative capacities. *Circ Res*. 2006;98:e20-5.

133.Tian H, McKnight SL and Russell DW. Endothelial PAS domain protein 1 (EPAS1), a transcription factor selectively expressed in endothelial cells. *Genes Dev*. 1997;11:72-82.

134.Saharinen P, Eklund L and Alitalo K. Therapeutic targeting of the angiopoietin-TIE pathway. *Nat Rev Drug Discov*. 2017;16:635-661.

135.Benezra R, Rafii S and Lyden D. The Id proteins and angiogenesis. *Oncogene*. 2001;20:8334-41.

136.Coulthard MG, Morgan M, Woodruff TM, Arumugam TV, Taylor SM, Carpenter TC, Lackmann M and Boyd AW. Eph/Ephrin signaling in injury and inflammation. *Am J Pathol*. 2012;181:1493-503.

137.Dallinga MG, Yetkin-Arik B, Kayser RP, Vogels IMC, Nowak-Sliwinska P, Griffioen AW, van Noorden CJF, Klaassen I and Schlingemann RO. IGF2 and IGF1R identified as novel tip cell genes in primary microvascular endothelial cell monolayers. *Angiogenesis*. 2018;21:823-836.

138.Kovacevic I, Muller M, Kojonazarov B, Ehrke A, Randriamboavonjy V, Kohlstedt K, Hindemith T, Schermuly RT, Fleming I, Hoffmeister M and Oess S. The F-BAR Protein NOSTRIN Dictates the Localization of the Muscarinic M3 Receptor and Regulates Cardiovascular Function. *Circ Res*. 2015;117:460-9.

139.Inoue M, Ishida T, Yasuda T, Toh R, Hara T, Cangara HM, Rikitake Y, Taira K, Sun L, Kundu RK, Quertermous T and Hirata K. Endothelial cell-selective adhesion molecule modulates atherosclerosis through plaque angiogenesis and monocyte-endothelial interaction. *Microvasc Res*. 2010;80:179-87.

140.Chen LC, Chen CC, Liang Y, Tsang NM, Chang YS and Hsueh C. A novel role for TNFAIP2: its correlation with invasion and metastasis in nasopharyngeal carcinoma. *Mod Pathol*. 2011;24:175-84.

141.Doddapattar P, Dhanesha N, Chorawala MR, Tinsman C, Jain M, Nayak MK, Staber JM and Chauhan AK. Endothelial Cell-Derived. Von Willebrand Factor, But Not Platelet-Derived, Promotes Atherosclerosis in Apolipoprotein E-Deficient Mice. *Arterioscler Thromb Vasc Biol*. 2018;38:520-528.

142.Stenmark KR, Frid M and Perros F. Endothelial-to-Mesenchymal Transition: An Evolving Paradigm and a Promising Therapeutic Target in PAH. *Circulation*. 2016;133:1734-7.

143.Hardy SA, Mabotuwana NS, Murtha LA, Coulter B, Sanchez-Bezanilla S, Al-Omary MS, Senanayake T, Loering S, Starkey M, Lee RJ, Rainer PP, Hansbro PM and Boyle AJ. Novel role of extracellular matrix protein 1 (ECM1) in cardiac aging and myocardial infarction. *PLoS One*. 2019;14:e0212230.

144.Liu H, Kennard S and Lilly B. NOTCH3 expression is induced in mural cells through an autoregulatory loop that requires endothelial-expressed JAGGED1. *Circ Res*. 2009;104:466-75.

145.Goldsmith EC, Zhang X, Watson J, Hastings J and Potts JD. The collagen receptor DDR2 is expressed during early cardiac development. *Anat Rec (Hoboken)*. 2010;293:762-9.

146.Lilly AJ, Mazan A, Scott DA, Lacaud G and Kouskoff V. SOX7 expression is critically required in FLK1-expressing cells for vasculogenesis and angiogenesis during mouse embryonic development. *Mech Dev*. 2017;146:31-41.

147.Romagnani P, Lasagni L, Annunziato F, Serio M and Romagnani S. CXC chemokines: the regulatory link between inflammation and angiogenesis. *Trends Immunol*. 2004;25:201-9.
