# Supplementary figures and images for: Cardiovascular disease risk factors induce mesenchymal features and senescence in mouse cardiac endothelial cells
Source: eLife. 2021 Mar 4;10:e62678. doi: 10.7554/eLife.62678 (PMC8043751; doi:10.7554/eLife.62678)

## Slide 1
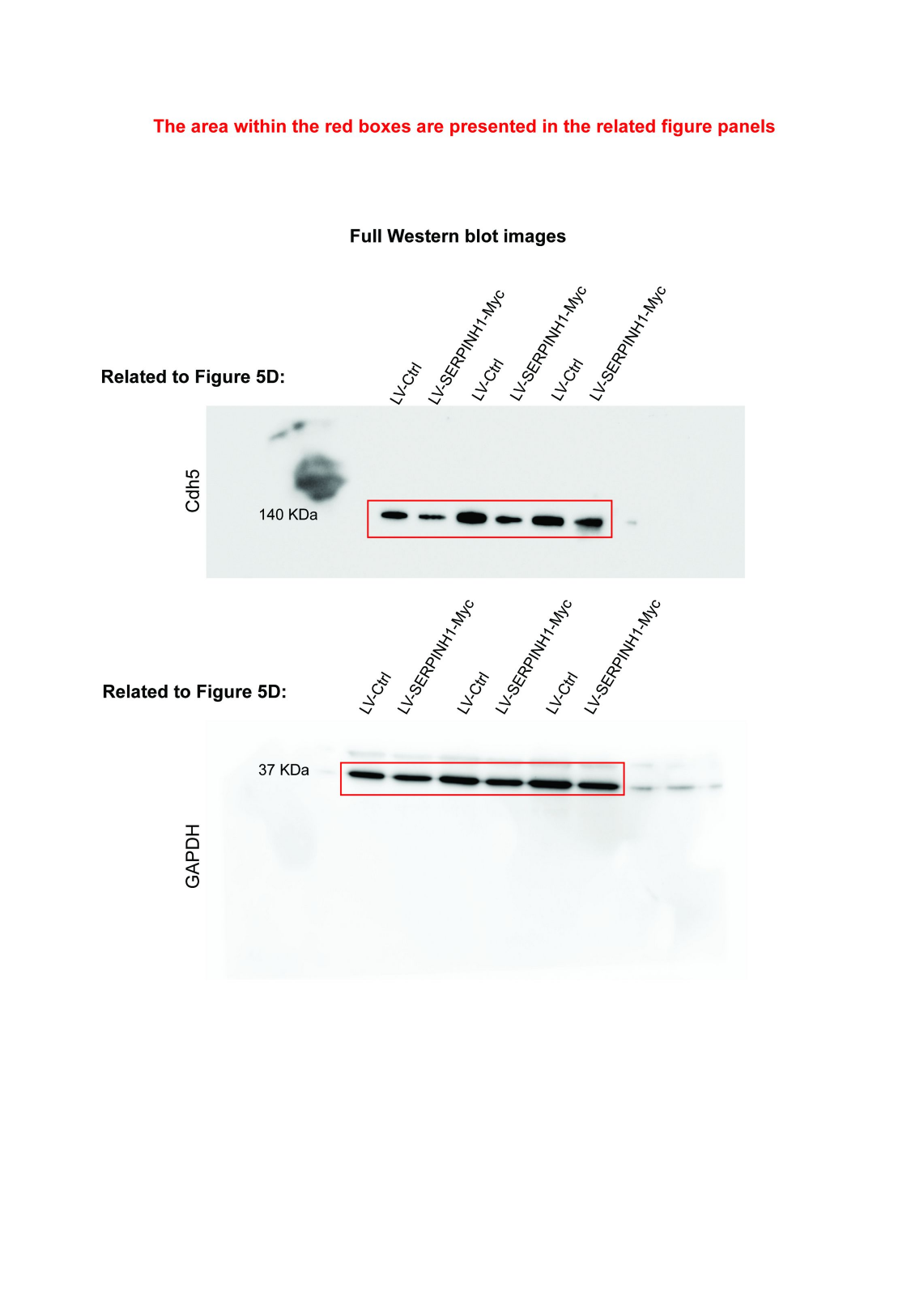

Supplement: Figure 5—source data 2. [file elife-62678-fig5-data2.pptx]

## Slide 1
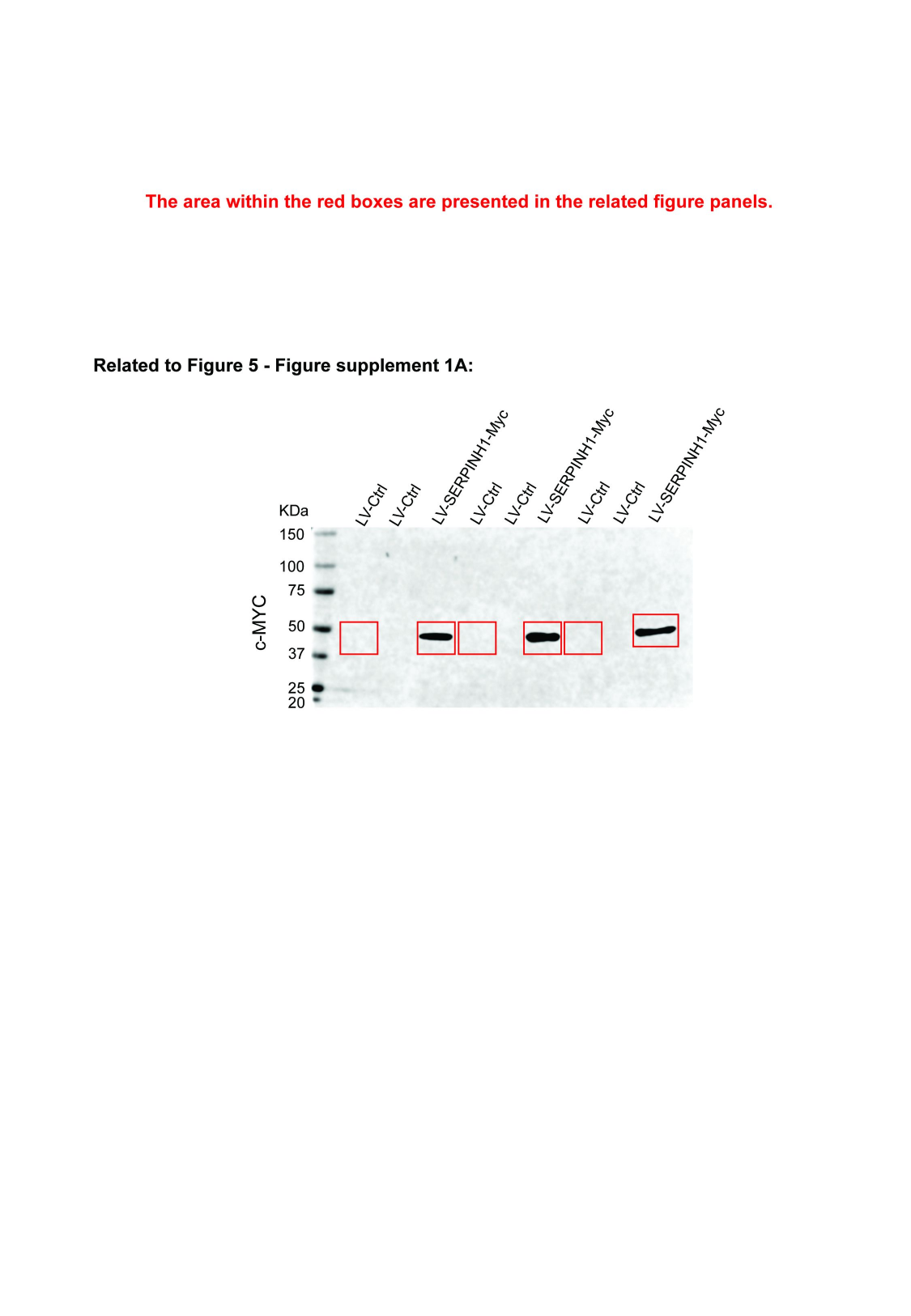

Supplement: Figure 5—figure supplement 1—source data 1. [file elife-62678-fig5-figsupp1-data1.pptx]
